# Psychiatric liaison team memory pathway: does it achieve the standards set out in NICE clinical guideline 97?

**DOI:** 10.1192/bjo.2021.913

**Published:** 2021-06-18

**Authors:** Manouri Senaratne, Afshan Jabeen, Alisha Hyams, Carol Deary, Sarah Stathers, Angus Brown, Eleanor Stebbings, Sunita Sahu, Roger Smyth, Hilary Kinsler, Stephen O'Connor, Andrew Wells, Ross Overshott, Kehinde Junaid, Aparna Mordekar, Jenny Humphries, Karen James, Shweta Mittal, Sarita Dasari, Hugh Grant-Peterkin, Niall Campbell, George Tadros, Elizabeth Sampson, Robert West

**Affiliations:** 1 South West Yorkshire Partnership NHS Foundation Trust; 2 South West Yorkshire Partnership NHS Foundation Trust; Cumbria, Northumbria and Tees Valley NHS Foundation Trust; 3 Cambridgeshire and Peterborough NHS Foundation Trust; 4 South London and Maudsley NHS Foundation Trust; 5 Oxleas NHS Foundation Trust; 6 NHS Lothian; 7 North East London NHS Foundation Trust; 8 Greater Manchester Mental Health NHS Foundation Trust; 9 Nottinghamshire Healthcare NHS Foundation Trust; 10 Sheffield Health and Social Care NHS Foundation Trust; 11 Avon and Wiltshire Mental Health Partnership NHS Trust; 12 Hywel Dda University Health Board; 13 Humber Teaching NHS Foundation Trust; 14 East London NHS Foundation Trust; 15 Chesire and Wirral NHS Foundation Trust; 16 Birmingham and Solihull Mental Health Foundation Trust; 17 Barnet, Enfield and Haringey Mental Health Trust and University College London; 18 University of Leeds

## Abstract

**Aims:**

Early assessment, diagnosis and management for people living with dementia is essential, both for the patient and their carers. We recognised delays in established local pathways when patients had unplanned acute hospital admissions preventing them from attending memory diagnostic appointments. The Psychiatric Liaison Team (PLT) Memory Pathway was introduced as we had the skills and expertise to resume the process and to find new undetected patients.

Our aim was to determine how well the newly implemented PLT Memory Pathway follows the standards outlined in the National Institute of Health & Care Excellence (NICE) Clinical Guideline 97 (CG97): Assessment, management and support for people living with dementia and their carers.

**Method:**

A retrospective analysis of all PLT referrals from July 2018 to February 2020 (20 months) was performed to identify patients on the community memory pathway and those with possible undetected cognitive impairment. Data were collected from electronic patient records which included demographics, primary and collateral history, cognitive testing and imaging, dementia type among others. Results were analysed using Microsoft Excel.

**Result:**

41 patients were included (59% female). 80% of patients were referred for memory problems or confusion. 63% had previous referrals to a memory service and was on the community memory pathway at the time of the referral. 34% were on anticholinergic medication but in only 14% were this documented as reviewed. 100 % were offered and had head imaging. A finding worthy of note was the absence of any from the ethnic minority background. 63% of patients were given a memory diagnosis and 34% had anti-dementia medication started. Patients’ families were made aware of the diagnosis in 83% of cases, due to the absence of next of kin details in the patient record. Primary Care was made aware in 100% of cases; post-diagnostic support was 100%.

**Conclusion:**

The PLT is well placed to bridge the service gap between the acute care trust and established community memory services when dealing with patients with dementia. A dedicated Memory Pathway has helped to close this gap and adherence to NICE CG97 standards was good, but there is room for improvement. A particular focus will be on improving documentation of anticholinergic medication review and exploration for the absence of ethnic minority patients. Aiming to achieve 100% family involvement is also recommended.

This study has been submitted to the Royal College of Psychiatrists' Faculty of Old Age Annual Conference 2021.

